# P-1978. Neuropsychiatric Events Linked to Fluoroquinolones: Signal Detection and Trend Analysis in the FAERS Database

**DOI:** 10.1093/ofid/ofaf695.2145

**Published:** 2026-01-11

**Authors:** Ashin Siby, Manu Mathew, Jose T John

**Affiliations:** Durdans Hospital, Colombo, Western Province, Sri Lanka; Durdans Hospital, Colombo, Western Province, Sri Lanka; Durdans Hospital, Colombo, Western Province, Sri Lanka

## Abstract

**Background:**

Fluoroquinolones are widely prescribed for respiratory, urinary, and gastrointestinal infections. Despite their clinical utility, emerging evidence has associated them with neuropsychiatric adverse events (AEs), ranging from mild confusion to hallucinations and suicidality. This study aimed to evaluate the signal strength and temporal trends of neuropsychiatric AEs associated with fluoroquinolone use in real-world settings using the U.S. FDA Adverse Event Reporting System (FAERS) database.

**Figure:**
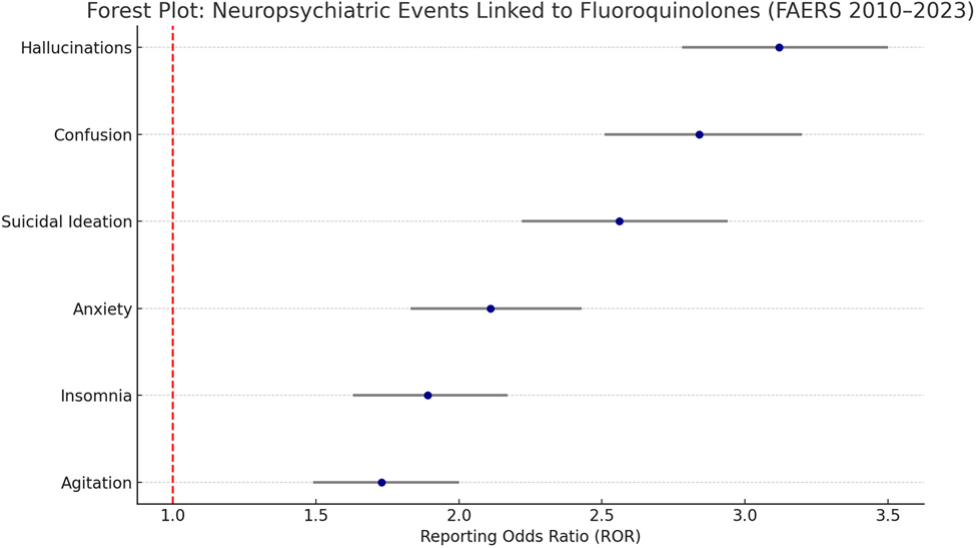
Forest Plot: Neuropsychiatric Events Linked to Fluoroquinolones (FAERS 2010–2023) This forest plot displays Reporting Odds Ratios (RORs) with 95% confidence intervals for neuropsychiatric adverse events associated with fluoroquinolone use. Strong signals were observed for hallucinations (ROR: 3.12), confusion (ROR: 2.84), and suicidal ideation (ROR: 2.56), emphasizing the need for cautious prescribing in neurologically vulnerable populations.

**Methods:**

A retrospective pharmacovigilance study was conducted using FAERS reports from January 2010 to December 2023. Reports that identified ciprofloxacin, levofloxacin, moxifloxacin, or ofloxacin as the primary suspect drugs were included. Neuropsychiatric-related Preferred Terms (PTs) were extracted from MedDRA, including insomnia, agitation, anxiety, depression, hallucinations, confusion, and suicidal ideation. Disproportionality analysis using Reporting Odds Ratios (RORs) and 95% confidence intervals (CIs) was performed. A signal was defined as ROR lower CI >1 and ≥3 cases. Year-wise trend analysis was conducted to assess reporting frequency.

**Results:**

A total of 8,794 neuropsychiatric AE reports were associated with fluoroquinolones. Ciprofloxacin accounted for the highest number (4,235), followed by levofloxacin (2,961) and moxifloxacin (1,126). Strong signals were identified for hallucinations (ROR: 3.12, 95% CI: 2.78–3.50), confusion (ROR: 2.84), and suicidal ideation (ROR: 2.56). Ciprofloxacin and levofloxacin showed the most pronounced associations. Reporting frequency for neuropsychiatric AEs rose markedly between 2016 and 2020, possibly reflecting heightened regulatory warnings and clinical awareness.

**Conclusion:**

This FAERS-based analysis confirms a consistent safety signal linking fluoroquinolones with serious neuropsychiatric adverse events. The trend suggests increasing awareness but also reinforces the need for careful patient evaluation, particularly among elderly individuals or those with preexisting CNS conditions. Real-world pharmacovigilance data remain vital for guiding antibiotic safety and stewardship.

**Disclosures:**

All Authors: No reported disclosures

